# Social Factors Predictive of Intensive Care Utilization in Technology-Dependent Children, a Retrospective Multicenter Cohort Study

**DOI:** 10.3389/fped.2021.721353

**Published:** 2021-09-13

**Authors:** Katherine N. Slain, Amie Barda, Peter J. Pronovost, J. Daryl Thornton

**Affiliations:** ^1^Department of Pediatrics, University Hospitals Rainbow Babies & Children's Hospital, Cleveland, OH, United States; ^2^Case Western Reserve University School of Medicine, Cleveland, OH, United States; ^3^Department of Anesthesiology and Critical Care Medicine, University Hospitals Cleveland Medical Center, Cleveland, OH, United States; ^4^Center for Reducing Health Disparities, MetroHealth Campus of Case Western Reserve University, Cleveland, OH, United States; ^5^Center for Population Health Research, MetroHealth Campus of Case Western Reserve University, Cleveland, OH, United States

**Keywords:** healthcare disparities, intensive care units, pediatric, tracheostomy, gastrostomy, race

## Abstract

**Objective:** Technology-dependent children with medical complexity (CMC) are frequently admitted to the pediatric intensive care unit (PICU). The social risk factors for high PICU utilization in these children are not well described. The objective of this study was to describe the relationship between race, ethnicity, insurance status, estimated household income, and PICU admission following the placement of a tracheostomy and/or gastrostomy (GT) in CMC.

**Study Design:** This was a retrospective multicenter study of children <19 years requiring tracheostomy and/or GT placement discharged from a hospital contributing to the Pediatric Health Information System (PHIS) database between January 2016 and March 2019. Primary predictors included estimated household income, insurance status, and race/ethnicity. Additional predictor variables collected included patient age, sex, number of chronic complex conditions (CCC), history of prematurity, and discharge disposition following index hospitalization. The primary outcome was need for PICU readmission within 30 days of hospital discharge. Secondary outcomes included repeated PICU admissions and total hospital costs within 1 year of tracheostomy and/or GT placement.

**Results:** Patients requiring a PICU readmission within 30 days of index hospitalization for tracheostomy or GT placement accounted for 6% of the 20,085 included subjects. In multivariate analyses, public insurance [OR 1.28 (95% C.I. 1.12–1.47), *p* < 0.001] was associated with PICU readmission within 30 days of hospital discharge while living below the federal poverty threshold (FPT) was associated with a lower odds of 30-day PICU readmission [OR 0.7 (95% C.I. 0.51–0.95), *p* = 0.0267]. Over 20% (*n* = 4,197) of children required multiple (>1) PICU admissions within one year from index hospitalization. In multivariate analysis, Black children [OR 1.20 (95% C.I. 1.10–1.32), *p* < 0.001] and those with public insurance [OR 1.34 (95% C.I. 1.24–1.46), *p* < 0.001] had higher odds of multiple PICU admissions. Social risk factors were not associated with total hospital costs accrued within 1 year of tracheostomy and/or GT placement.

**Conclusions:** In a multicenter cohort study, Black children and those with public insurance had higher PICU utilization following tracheostomy and/or GT placement. Future research should target improving healthcare outcomes in these high-risk populations.

## Introduction

Nearly one-third of health care costs for children are attributed to the one percent of children with medical complexity (CMC), in part due to their need for frequent hospitalizations (1, 2). In the United States (US), a subset of technology-dependent CMC rely upon life-sustaining medical technologies, including gastrointestinal and respiratory devices (3). Because of their medical fragility and the complication rate of medical devices, technology-dependent children are at risk for high hospital utilization, including frequent admissions and need for intensive care. An estimated 20% of admissions to a US pediatric intensive care unit (PICU) are children with a tracheostomy or gastrostomy tube (GT) (4).

The risk factors for PICU admission in technology-dependent children have not been well described but are likely similar to risk factors for general hospital admission including younger age and higher medical complexity (5). There is some evidence to suggest that technology-dependent children of minority race and those living in poverty with inadequate environmental conditions are at risk for repeated emergency department visits, prolonged hospital stays, and hospital readmission, and have a higher mortality (6–9).

The poor outcomes experienced by technology-dependent children could reflect the challenge families experience in caring for CMC at home (6). Families consistently report difficulties in both navigating the healthcare system and unmet health needs including lack of subspecialty care (10, 11). These challenges are likely much worse in socially and economically disadvantaged families. Optimal health outcomes for technology-dependent children require sophisticated care coordination centered within the medical home (10).

The relationship between poor living conditions and poor health in children is well documented (11). The impact of social factors including poverty, racism, inadequate insurance coverage, inadequate housing, food insecurity, and insufficient healthcare access may be most detrimental in technology-dependent children and mitigating the risk of PICU utilization requires understanding these risk factors. Yet little is known about the relationship between social factors and PICU utilization. Therefore, the primary objective of this study was to use a multicenter database of US children's hospitals to test the hypothesis that social factors including estimated household income, insurance status, and race predict high PICU utilization, defined as need for PICU readmission within 30-days of discharge from the hospital following initial placement of tracheostomy or GT. Secondary outcomes included repeated PICU admissions, and total hospital costs within 1 year following tracheostomy or GT placement.

## Methods

### Data Source

Data for this retrospective, multicenter cohort study was obtained from the Pediatric Health Information System (PHIS) a quality-controlled database of over 45 tertiary children's hospitals located in non-competing markets across the US associated with the Children's Hospital Association (Lenexa, KS). PHIS accounts for 15% of US pediatric hospitalizations and contains comprehensive patient-level data including demographics, admission and discharges dates, *International Classification of Diseases, Tenth Revisions, Clinical Modification* (ICD-10-CM, ICD-10-PCS) diagnosis and procedure codes, billing data, and disposition data (12). Although the database is de-identified, encrypted patient identification numbers allow investigators to track subjects across multiple hospitalizations. The study protocol was reviewed by the University Hospitals Institutional Review Board and deemed exempt from ethical approval and oversight (STUDY20201822).

### Study Population

Children <19 years of age with a procedure code for a tracheostomy (*ICD-10-PCS*: 0B110F4, 0B113F4, 0B114F4) or GT (*ICD-10-PCS*: 0DH60UZ, 0DH63UZ, 0DH64UZ) discharged from a PHIS hospital from January 2016 through March 2019 were included in the study cohort as index hospitalizations and data collection continued for 365 days after discharge from index hospitalization, until March 2020 (6, 8).

### Data Collection and Study Protocol

The primary outcome of interest was PICU utilization within 30 days of discharge from the index hospitalization. PICU utilization was defined by presence of the PICU “flag”, which identifies PICU level of care (excluding neonatal ICU care) through billing codes. Other outcomes examined included repeated (>1) PICU admissions and total accrued hospital costs within 365 days of discharge from index hospitalization, hospital length of stay (LOS) at index admission, 30-day hospital readmission, and discharge disposition for children requiring repeated hospital admissions within the one-year observation period. Because hospital readmissions and total hospital costs were outcomes of interest, children who died on the index admission or subsequent readmissions, and children with missing cost data were excluded.

**Figure 1:**
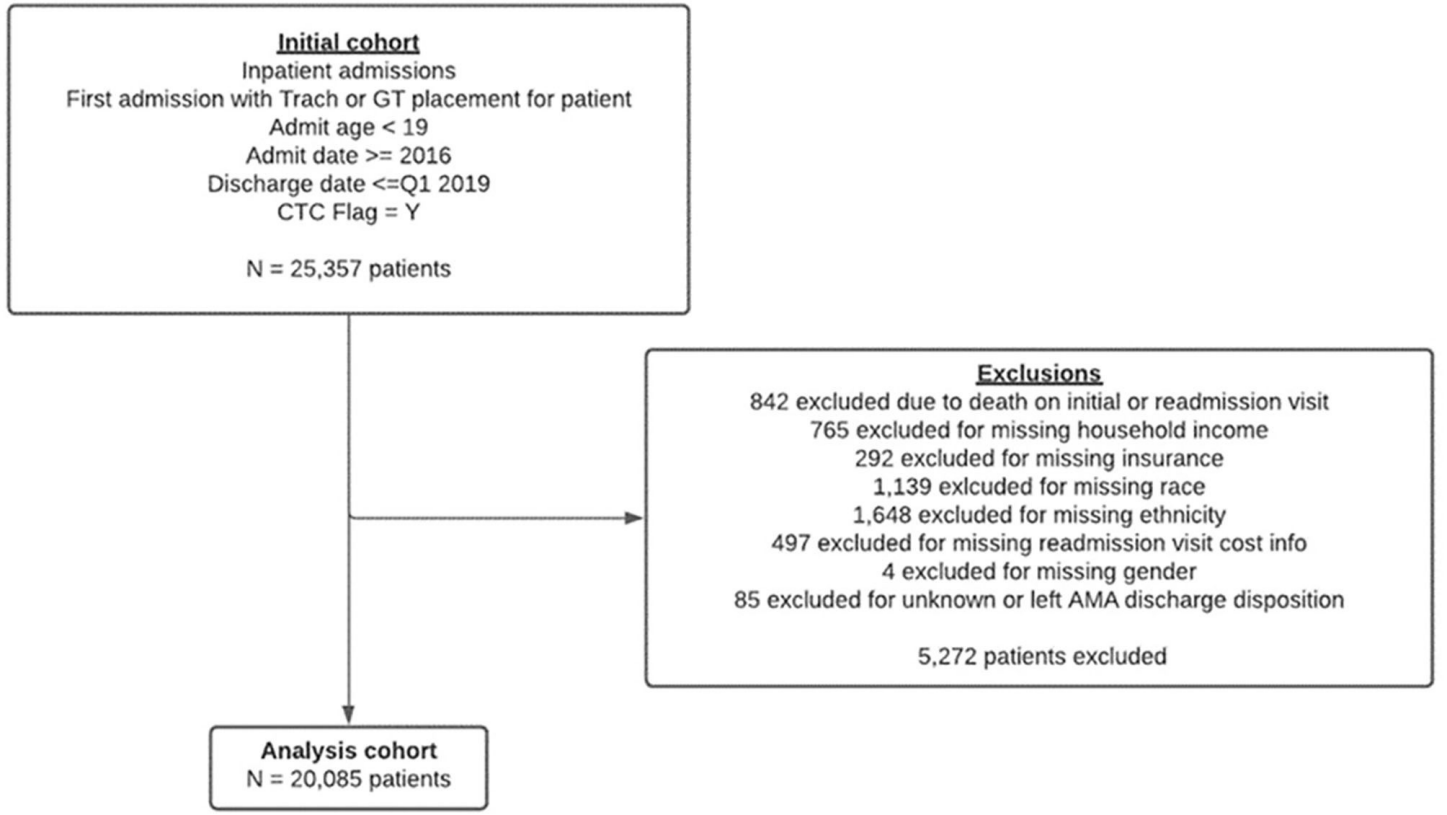
Patient flow diagram showing inclusion and exclusion criteria.

The primary sociodemographic predictors included patient income, race/ethnicity, and insurance. The median household income provided by PHIS was obtained by linking patients' ZIP codes to data from the US census bureau. The 2016 federal poverty threshold (FPT) for a family of four with two children was $24,339 (13). For this study, subjects were categorized into four groups based on the FPT and its percentage multiples, which are commonly used as eligibility criterion for various federal programs (14). Race and ethnicity were categorized White, Black, Asian, Hispanic or Latino, or “other,” which included Pacific Islander, American Indian or Alaskan Native, other race, or mixed race. Insurance status was categorized into private, public or “other,” which included patients classified as charity, self-pay, no hospital bill associated with subject, or “other payor.” Additional predictor variables for the outcomes of interest included age, analyzed as a categorical variable, sex, number of chronic complex conditions (CCC) using the classification system proposed by Feudtner et al., history of prematurity or low birthweight, and discharge disposition at index hospitalization, categorized as discharge to home, discharge to home with health services, or discharge to another healthcare facility (Supplementary Table 1) (15, 16). Children with missing predictor variables including income, race/ethnicity, insurance, sex, or discharge disposition were excluded (Figure 1).

### Statistical Analysis

The demographics of study subjects at index hospitalization were summarized using descriptive statistics. All variables were categorical and presented as frequencies and percentages. Univariate associations between predictor variables and outcomes of interest including PICU readmission within 30 days of index hospitalization, >1 PICU admission within 365 days of index hospitalization, 30-day hospital readmission, discharge disposition at index admission, and discharge disposition after repeated admissions, were analyzed using chi-square tests. Variables associated with these outcomes in univariate analysis (*p* < 0.05) and the primary predictors of interest (race, ethnicity, median household income and insurance status) regardless of statistical significance, were included in multivariate logistic regression analyses. For the continuous outcomes of hospital LOS at index admission and total hospital costs within 365 days of index hospitalization, we observed that these outcomes were positively skewed and violated the normality assumption. Therefore, we assessed the relationship between predictor variables and the continuous outcomes using univariate and multivariate generalized linear models using the gamma distribution with a log-link. Presence of multi-collinearity between the predictors was checked by the variation inflation factor [VIF] and no substantial multi-collinearity (defined as VIF > 5) was observed. For the outcome of discharge disposition after repeat hospital admissions, we included the population of patients who experienced a 365-day readmission, excluding those with an unknown discharge disposition on subsequent discharges (*n* = 22), for a final cohort of 14,959 patients. For patients experiencing multiple discharges during the one-year observation period, we categorized them based on the highest level of care used (Healthcare facility > Home with health services > Home). In the multinomial logistic regression analysis, we used “Home” as the comparison category. For all analyses, *p* < 0.05 were considered statistically significant and odds ratios were considered significant if the 95% confidence intervals did not include 1. All analyses were conducted using R software, version 4.0.3.

## Results

There were 20,085 children <19 years of age who were admitted for a tracheostomy or GT placement to one of 49 PHIS hospitals during the study period. Demographics and clinical characteristics at index hospitalization are found in Table 1. Most children were <1 year of age (58%), White (62%), and had public insurance (63%). Over two-thirds of children lived in a low-income ZIP code where the median household income was <200% of the FPT. Nearly all children (*n* = 19,876) were classified as having a chronic complex condition, with technology-dependence (93%), gastrointestinal (89%), and neurologic/neuromuscular (34%) being the most common categories (Supplementary Table 2). Only 15% of children in this cohort were discharged home with health services, and there were differences in patient demographics and clinical characteristics based on discharge disposition (Supplementary Tables 1, 3).

**Table 1:** Patient demographics and clinical characteristics at index admission in a cohort of technology-dependent children.

**Characteristic**	**Entire cohort *n* = 20,085**
Admit age	
<1 month	6,454 (32%)
1–12 months	5,176 (26%)
1–2 years	1,880 (9.4%)
2–5 years	2,221 (11%)
5–11 years	2,128 (11%)
>11 years	2,226 (11%)
Male	10,953 (55%)
Hispanic or Latino ethnicity	3,908 (19%)
Race	
White	12,418 (62%)
Black	3,826 (19%)
Asian	702 (3.5%)
Other	3,139 (16%)
Median household income (% FPT)	
>$48,678 (>200%)	6,396 (32%)
$36,509–$48,678 (150–200%)	6,288 (31%)
$24,339–$36,509 (100–150%)	6,487 (32%)
< $24,339 (<100%)	914 (4.6%)
Insurance	
Private	7,001 (35%)
Public	12,729 (63%)
Other	355 (1.8%)
Number of complex chronic conditions	
1 or fewer	656 (3.3%)
2 to 4	14,307 (71%)
5 or more	5,122 (26%)
History of prematurity/low birthweight	3,232 (16%)
Procedure received	
GT	16,822 (84%)
Tracheostomy	1,509 (7.5%)
Both	1,754 (8.7%)
Discharge disposition	
Home	15,444 (77%)
Home with health services	3,011 (15%)
Healthcare facility	1,630 (8.1%)

*Statistics presented as n (%); FPT, federal poverty threshold; GT, gastrostomy tube*.

Only 6% (*n* = 1,264) of children required a PICU readmission within 30 days of discharge from the index hospitalization (Table 2). In an adjusted multivariate model, public insurance was associated with higher odds of 30-day PICU readmission [OR 1.28 (95% C.I. 1.12–1.47), *p* < 0.001], while living below the FPT was associated with a lower odds of 30-day PICU readmission [OR 0.7 (95% C.I. 0.51–0.95), *p* = 0.0267].

**Table 2:** Univariate and multivariate analysis of characteristics associated with a 30-day PICU readmission in a cohort of technology-dependent children.

**Characteristic**	**Univariate analysis**			**Multivariate analysis**		
	**Not readmitted to PICU**	**Readmitted to PICU**	***p*-value**	**OR**	**95% CI**	***p*-value**
	***n* = 18,821**	***n* = 1,264**				
Admit age			<0.001			
<1 month	5,938 (32%)	516 (41%)		*Reference*		
1–12 months	4,816 (26%)	360 (28%)		0.98	0.84, 1.13	0.8
1–2 years	1,782 (9.5%)	98 (7.8%)		0.79	0.63, 0.99	0.049
2–5 years	2,117 (11%)	104 (8.2%)		0.67	0.53, 0.84	<0.001
5–11 years	2,043 (11%)	85 (6.7%)		0.53	0.41, 0.67	<0.001
>11 years	2,125 (11%)	101 (8.0%)		0.55	0.44, 0.69	<0.001
Sex			0.026			
Male	10,225 (54%)	728 (58%)		*Reference*		
Female	8,596 (46%)	536 (42%)		0.89	0.79, 0.99	0.041
Ethnicity			0.4			
Not Hispanic or Latino	15,171 (81%)	1,006 (80%)		*Reference*		
Hispanic or Latino	3,650 (19%)	258 (20%)		1.02	0.87, 1.20	0.8
Race			0.004			
White	11,696 (62%)	722 (57%)		*Reference*		
Black	3,551 (19%)	275 (22%)		1.08	0.93, 1.26	0.3
Asian	657 (3.5%)	45 (3.6%)		1.15	0.83, 1.57	0.4
Other	2,917 (15%)	222 (18%)		1.14	0.96, 1.34	0.13
Median household income (% FPT)			0.2			
>$48,678 (>200%)	6,018 (32%)	378 (30%)		*Reference*		
$36,509–$48,678 (150–200%)	5,879 (31%)	409 (32%)		1.01	0.87, 1.18	0.9
$24,339–$36,509 (100–150%)	6,059 (32%)	428 (34%)		0.97	0.83, 1.12	0.7
< $24,339 (<100%)	865 (4.6%)	49 (3.9%)		0.7	0.51, 0.95	0.027
Insurance			<0.001			
Private	6,632 (35%)	369 (29%)		*Reference*		
Public	11,859 (63%)	870 (69%)		1.28	1.12, 1.47	<0.001
Other	330 (1.8%)	25 (2.0%)		1.43	0.91, 2.16	0.1
Number of complex chronic conditions			<0.001			
1 or fewer	632 (3.4%)	24 (1.9%)		*Reference*		
2–4	13,621 (72%)	686 (54%)		1.19	0.80, 1.85	0.4
5 or more	4,568 (24%)	554 (44%)		2.12	1.42, 3.33	<0.001
History of prematurity/low birthweight			<0.001			
No	15,844 (84%)	1009 (80%)		*Reference*		
Yes	2,977 (16%)	255 (20%)		1.04	0.89, 1.20	0.6
Procedure received			<0.001			
GT	15,965 (85%)	857 (68%)		*Reference*		
Tracheostomy	1,335 (7.1%)	174 (14%)		2.28	1.89, 2.74	<0.001
Both	1,521 (8.1%)	233 (18%)		1.93	1.61, 2.29	<0.001
Discharge disposition			<0.001			
Home	14,576 (77%)	868 (69%)		*Reference*		
Home with health services	2,771 (15%)	240 (19%)		1.22	1.04, 1.42	0.011
Healthcare facility	1,474 (7.8%)	156 (12%)		1.13	0.93, 1.38	0.2

*PICU, pediatric intensive care unit; OR, odds ratio; CI, confidence interval; FPT, federal poverty threshold; GT, gastrostomy tube*.

Over 20% (*n* = 4,197) of children required multiple (>1) PICU admissions within one year from the index hospitalization (Table 3). In univariate analysis, there were differences in age, race, insurance, number of chronic complex conditions, history of prematurity or low birthweight, procedure received, and discharge disposition between children who did and did not require multiple PICU hospitalizations. In multivariate analysis, Black children [OR 1.20 (95% C.I. 1.10–1.32), *p* < 0.001] and those with public insurance [OR 1.34 (95% C.I. 1.24–1.46), *p* < 0.001] had higher odds of multiple PICU admissions. Estimated household income level was not an independent predictor of high PICU utilization in this cohort of children.

**Table 3:** Univariate and multivariate analysis of characteristics associated with >1 365-day PICU readmission in a cohort of technology-dependent children.

	**Univariate analysis**			**Multivariate analysis**		
**Characteristic**	**≤1 PICU Readmission**	**>1 PICU Readmission**	***p*-value**	**OR**	**95% CI**	***p*-value**
	***n* = 15,888**	***n* = 4,197**				
Admit age			<0.001			
<1 month	5,231 (33%)	1,223 (29%)		*Reference*		
1–12 months	3,963 (25%)	1,213 (29%)		1.33	1.22, 1.46	<0.001
1–2 years	1,398 (8.8%)	482 (11%)		1.43	1.26, 1.62	<0.001
2–5 years	1,723 (11%)	498 (12%)		1.26	1.11, 1.42	<0.001
5–11 years	1,717 (11%)	411 (9.8%)		1.09	0.96, 1.24	0.2
>11 years	1,856 (12%)	370 (8.8%)		0.98	0.86, 1.12	0.8
Sex			0.14			
Male	8,707 (55%)	2,246 (54%)				
Female	7,181 (45%)	1,951 (46%)				
Ethnicity			0.088			
Not Hispanic or Latino	12,836 (81%)	3,341 (80%)		*Reference*		
Hispanic or Latino	3,052 (19%)	856 (20%)		1.07	0.97, 1.17	0.2
Race			<0.001			
White	9,897 (62%)	2,521 (60%)		*Reference*		
Black	2,910 (18%)	916 (22%)		1.2	1.10, 1.32	<0.001
Asian	575 (3.6%)	127 (3.0%)		0.89	0.73, 1.08	0.3
Other	2,506 (16%)	633 (15%)		0.93	0.84, 1.03	0.2
Median household income (% FPT)			0.2			
>$48,678 (>200%)	5,082 (32%)	1,314 (31%)		*Reference*		
$36,509–$48,678 (150–200%)	4,985 (31%)	1,303 (31%)		0.95	0.87, 1.04	0.3
$24,339–$36,509 (100–150%)	5,124 (32%)	1,363 (32%)		0.94	0.86, 1.03	0.2
< $24,339 (<100%)	697 (4.4%)	217 (5.2%)		1.09	0.92, 1.29	0.3
Insurance			<0.001			
Private	5,762 (36%)	1,239 (30%)		*Reference*		
Public	9,843 (62%)	2,886 (69%)		1.34	1.24, 1.46	<0.001
Other	283 (1.8%)	72 (1.7%)		1.26	0.96, 1.64	0.093
Number of complex chronic conditions			<0.001			
1 or fewer	529 (3.3%)	127 (3.0%)		*Reference*		
2–4	11,177 (70%)	3,130 (75%)		1.25	1.03, 1.53	0.027
5 or more	4,182 (26%)	940 (22%)		1.23	1.00, 1.53	0.051
History of prematurity/low birthweight			0.047			
No	13,374 (84.2%)	3479 (82.9%)		*Reference*		
Yes	2514 (16%)	718 (17%)		1.11	1.01, 1.22	0.03
Procedure received			<0.001			
GT	13,043 (82%)	3,779 (90%)		*Reference*		
Tracheostomy	1,318 (8.3%)	191 (4.6%)		0.57	0.48, 0.67	<0.001
Both	1,527 (9.6%)	227 (5.4%)		0.62	0.53, 0.72	<0.001
Discharge disposition			<0.001			
Home	12,013 (76%)	3,431 (82%)		*Reference*		
Home with health services	2,390 (15%)	621 (15%)		0.95	0.86, 1.05	0.4
Healthcare facility	1,485 (9.3%)	145 (3.5%)		0.42	0.35, 0.50	<0.001

*PICU, pediatric intensive care unit; OR, odds ratio; CI, confidence interval; FPT, federal poverty threshold; GT, gastrostomy tube*.

Race, ethnicity, household income and insurance were not associated with total hospital costs accrued in the 14,981 children who were admitted within one year of the index hospitalization following tracheostomy/GT placement (Table 4). In these children, multivariate models demonstrate that discharge disposition at index admission was the strongest predictor of discharge disposition upon subsequent hospitalizations; the impact of race/ethnicity, insurance status and estimated household income was negligible in comparison (Supplementary Table 4).

**Table 4:** Univariate and multivariate analysis of characteristics predictive of total hospital costs 1 year after tracheostomy/GT placement.

**Characteristic**	**Univariate analysis**			**Multivariate analysis**				
	**OR**	**95% CI**	***p*-value**	**OR**	**95% CI**	**% Change**	**95% CI**	***p*-value**
Admit age								
<1 month	*Reference*				*Reference*			
1–12 months	0.76	0.68, 0.85	<0.001	0.83	0.75, 0.94	−16.5	−25.4, −6.5	0.002
1–2 years	0.61	0.52, 0.71	<0.001	0.69	0.59, 0.81	−30.8	−40.8, −18.8	<0.001
2–5 years	0.66	0.57, 0.77	<0.001	0.74	0.63, 0.86	−26.3	−36.6, −14.0	<0.001
5–11 years	0.71	0.61, 0.83	<0.001	0.75	0.64, 0.88	−25	−35.8, −11.9	<0.001
>11 years	0.8	0.69, 0.94	0.006	0.78	0.67, 0.92	−21.5	−32.7, −8.0	0.003
Sex								
Male	*Reference*							
Female	1.01	0.92, 1.11	0.8					
Ethnicity								
Not Hispanic or Latino	*Reference*				*Reference*			
Hispanic or Latino	1.06	0.95, 1.19	0.3	1.01	0.90, 1.14	1.4	−9.7, 14.0	0.8
Race								
White	*Reference*				*Reference*			
Black	1	0.89, 1.13	>0.9	0.91	0.81, 1.02	−8.8	−18.6, 2.41	0.12
Asian	0.82	0.65, 1.07	0.13	0.81	0.65, 1.04	−18.5	−35.4, 4.5	0.093
Other	1.07	0.94, 1.21	0.3	0.99	0.87, 1.12	−1.2	−13.0, 12.5	0.9
Median household income (% FPT)								
>$48,678 (>200%)	*Reference*				*Reference*			
$36,509–$48,678 (150–200%)	1.02	0.91, 1.15	0.7	1.02	0.91, 1.13	1.6	−9.0, 13.5	0.8
$24,339–$36,509 (100–150%)	1.11	0.99, 1.24	0.077	1.08	0.97, 1.21	8.2	−3.4, 21.1	0.2
< $24,339 (<100%)	1.3	1.05, 1.63	0.02	1.2	0.97, 1.49	19.9	−2.8, 49.4	0.1
Insurance								
Private	*Reference*				*Reference*			
Public	1.03	0.93, 1.13	0.6	0.99	0.89, 1.09	−1.2	−10.6, 9.1	0.8
Other	0.89	0.64, 1.30	0.5	0.88	0.64, 1.26	−11.8	−36.2, 25.9	0.5
Number of complex chronic conditions								
1 or fewer	*Reference*				*Reference*			
2–4	1.11	0.85, 1.42	0.4	1.06	0.82, 1.34	5.8	−18.22, 34.4	0.7
5 or more	1.93	1.46, 2.51	<0.001	1.63	1.25, 2.10	63.3	24.8, 110.1	<0.001
History of prematurity/low birthweight								
No	*reference*							
Yes	0.99	0.88, 1.12	>0.9					
Procedure received								
GT	*Reference*				*Reference*			
Tracheostomy	1.59	1.34, 1.89	<0.001	1.22	1.03, 1.46	22.2	3.13, 45.9	0.021
Both	1.55	1.33, 1.81	<0.001	1.08	0.93, 1.27	8.4	−7.3, 27.5	0.3
Discharge disposition								
Home	*Reference*				*Reference*			
Home with health services	0.95	0.83, 1.08	0.4	0.88	0.78, 1.00	−11.5	−21.5, 0.1	0.047
Healthcare facility	2.09	1.76, 2.50	<0.001	1.82	1.53, 2.18	81.9	53.1, 117.6	<0.001

*PICU, pediatric intensive care unit; OR, odds ratio; CI, confidence interval; FPT, federal poverty threshold; GT, gastrostomy tube*.

Children who were Black or of “other” race, had low household incomes, and public insurance all had higher odds of longer hospital LOS during their index admission. In multivariable regression analyses, Black children experienced a 27% longer LOS compared to White children [OR 1.27 (95% C.I. 1.21–1.33), *p* < 0.001], children living below the poverty threshold had a 22% longer LOS compared to children living at >200% FPT [OR 1.22 (95% C.I. 1.11–1.33), *p* < 0.001], and children with public insurance had an 11% longer LOS than children with private insurance [OR 1.11 (95% C.I. 1.06–1.15), *p* < 0.001] (Supplementary Table 5).

Within 30 days of the index hospitalization, 34% (*n* = 6,754) of children were readmitted to the hospital (Supplementary Table 6). In the multivariable model, Hispanic ethnicity [OR 1.10 (95% C.I. 1.02–1.19), *p* = 0.018], Black race [OR 1.23 (95% C.I. 1.14–1.34), *p* < 0.001), and public insurance [OR 1.31 (95% C.I. 1.22–1.40), *p* < 0.001] were associated with higher odds of 30-day hospital readmission. In this model, children with an estimated household income between 100 and 150% FPT had lower odds of 30-day hospital readmission [OR 0.88 (95% C.I. 0.81–0.95), *p* < 0.001).

## Discussion

Technology-dependent children are at high risk for hospital admission and PICU utilization because of their medical fragility and complications inherent in their medical devices. The overall objective of this study was to understand if social risk factors for high PICU utilization existed in this patient population. To our knowledge, this is the first study to show differential PICU utilization based on insurance or race in a cohort of medically complex children.

There are several important observations made in this study. First, over two-thirds of children in this study were living in low-income households. While we did not show any clinically meaningful differences in PICU utilization based on estimated household income, the financial implications of supporting a medically complex child should nonetheless be considered when counseling families. Families consistently report financial and logistical complications in caring for medically complex children (17–20). In an observational study of 167 CMC with high rates of technology dependence, over half of families reported financial difficulties related to their child's health, and two-thirds reported that a family member had decreased work hours to care for their child (20). The burden on families is likely underestimated, as only 15% of children in our cohort were discharged home from the index hospital admission with skilled nursing care, a percentage that decreased to 8.7% following subsequent hospital discharges. In this study, we did not find socioeconomic factors to be strongly associated with discharge disposition, however. Prospective studies are needed to understand how family financial stress impacts the risk for acute illness in their technology-dependent child.

Second, compared to children of other races, Black children had unfavorable health outcomes following placement of a tracheostomy and/or GT. They had longer hospital LOS during the index hospitalization, 20% higher odds of 30-day hospital readmission, and 20% higher odds of multiple PICU admissions within one year of the index hospital stay. Differential quality of healthcare based on patient race is a well described pervasive flaw in our healthcare system (21). However, there are few studies investigating differences in the risk of pediatric critical illness or in PICU utilization based on race (22–24). There are numerous studies describing racial/ethnic disparities in neonatal intensive care. A recently published systematic review including 41 articles outlined pervasive differences in access to high quality healthcare systems, healthcare delivery, and neonatal patient outcomes, based on race (25). Studies show high risk Black infants are less likely to be referred to neonatal follow up clinics, and Black mothers report experiencing indifferent and ineffective communication from medical staff, impacting effective family-centered care (26–29). There is also adult literature to suggest that Black families are more likely to choose life-sustaining medical technology for their kin following cardiac arrest, trauma, and stroke, but suffer from more adverse events, worse patient experiences, and less improvement in functional outcomes when receiving home health care (30–35). Our study design precludes understanding why technology-dependent Black children have higher hospital and PICU utilization. But like the neonatal and adult populations, if hospital processes and healthcare systems are inadequate to educate, engage and assist Black families caring for medically complex children, and if the pervasive institutional racism isn't eliminated, then repeated admissions are likely (21).

Third, like Black race, public insurance was also associated with high PICU utilization including 30-day PICU readmission and multiple PICU admissions within a year of index hospitalization. Public insurance has repeatedly been associated with high hospital utilization in pediatric patients (24, 36, 37). Upon hospital discharge, parents of publicly insured children are more likely to make errors in discharge plans, perhaps related to the prevalence of low literacy in this population (38). In the community, many publicly insured children receive primary care in resource limited settings where high patient volumes may limit the face time with providers (39). Similarly, children with public insurance are less likely to have a medical home, and less likely to receive high-quality family-centered care, factors which will undoubtedly affect technology- dependent children (40, 41).

Fourth, it was unexpected that lower household income was not associated with higher PICU readmission rates. Multiple markers of low socioeconomic status, including household income, have consistently been associated with increased risk for repeated hospital admissions (42, 43). There are several reasons why income, race and insurance status may have had different influence on our study outcomes, which are likely reflective of the differential impact these variables have on population health, and study design limitations. “Social determinants of health” is an imprecise concept describing the interplay between inequalities in social position and inequalities in health. Defining social position is equally challenging and is conceptualized as a person's standing and power within social hierarchies, influenced by multiple factors including but not limited to, socioeconomic status, education, employment, gender, and race (44). Studies like this one reinforce that while race, income and insurance status are related, they are distinct measures that should be considered differently when designing interventions to mitigate their negative effect (45). Study design limitations also likely influenced our results. Hospitals within the PHIS database are primarily academic teaching centers located in large cities with higher rates of public insurance and lower median household income, compared to national data (46). Institution of medical technology is likely to occur at these tertiary hospitals, but low acuity readmissions to community hospitals for suburban and rural families will be missed by the PHIS database.

This study has additional limitations important to consider. First, we used an observational design in this study, so we are unable to establish a causal relationship between our exposure variables and PICU utilization. Second, it is a retrospective database study dependent upon high-quality database governance of participating tertiary academic children's hospitals. While generalizability of our findings may be limited, PHIS data is likely reflective of the care provided at over 200 children's hospitals and pediatric facilities within general hospitals that provide most critical care services for US children (12, 47, 48). Third, the identification of our study cohort with ICD codes could be inaccurate, however previous studies have suggested identification of tracheostomy and GT placement using this methodology has high sensitivity and specificity (49, 50). Fourth, the sociodemographic predictor variables may be inaccurate. Race and ethnicity may be misclassified in our patients, but prior work suggests that administrative data is adequate for identifying Hispanic and non-Hispanic ethnicity, and White and Black race (51). PHIS provides an estimated household income based on ZIP code, which is an imperfect, but likely accurate measure of socioeconomic status. Previous studies have identified ZIP codes as an adequate surrogate for neighborhood-based deprivation (52, 53). Fifth, readmission rates are likely underestimated, as children receiving care at hospitals not within the PHIS database are not included. Lastly, database studies do not provide any information on specific clinical reasons why children are readmitted, ability to identify clinical confounders is limited, and results can only be viewed as hypothesis generating.

## Conclusion

This large multicenter cohort study of technology-dependent children demonstrated differences in PICU and hospital utilization based on race and insurance status. While future prospective studies are necessary to validate our findings, they suggest that socially vulnerable children and their families are over-burdened by hospitalizations following institution of medical technology. Aggressive interventions to prevent multiple, high-risk, resource-heavy hospital admissions and readmissions must be started by anchor healthcare institutions, including the tertiary children's hospitals represented in this study, even before we understand why these differences exist.

## Data Availability Statement

The data analyzed in this study is subject to the following licenses/restrictions: Data for this article was obtained from the Pediatric Health Information Systems Database, which is available to members of the Children's Hospital Association (Lenexa, KS). Requests to access these datasets should be directed to childrenshospitals.org.

## Author Contributions

KS conceptualized and designed the study. AB completed the data curation and statistical analysis. PP and JT made contributions to the study analysis and interpretation of the results. KS drafted the initial manuscript. AB, PP, and JT reviewed and edited the manuscript. All authors gave final approval to the final version of the manuscript and agree to be accountable for all aspects of the work.

## Funding

Dr. Slain, Dr. Thornton, and the Center for Reducing Health Disparities are supported by NIH U54MD002265 and UL1TR002548.

## Conflict of Interest

The authors declare that the research was conducted in the absence of any commercial or financial relationships that could be construed as a potential conflict of interest.

## Publisher's Note

All claims expressed in this article are solely those of the authors and do not necessarily represent those of their affiliated organizations, or those of the publisher, the editors and the reviewers. Any product that may be evaluated in this article, or claim that may be made by its manufacturer, is not guaranteed or endorsed by the publisher.

## Abbreviations

CMC: children with medical complexity
US: United States
PICU: pediatric intensive care unit
GT: gastrostomy tube
PHIS: Pediatric Health Information System
ICD-10-CM and ICD-10-PCS, International Classification of Diseases, Tenth Revision: Clinical Modification and Procedure Classification System
LOS, length of stay, FPT: federal poverty threshold
CCC: chronic complex condition
IQR: interquartile range
OR: odds ratio
CI: confidence interval.
