# Supplementary material for: Social Factors Predictive of Intensive Care Utilization in Technology-Dependent Children, a Retrospective Multicenter Cohort Study
Source: Front Pediatr. 2021 Sep 13;9:721353. doi: 10.3389/fped.2021.721353 (PMC8475907; doi:10.3389/fped.2021.721353)
Supplement: Supplementary file 1 [file Table_1.DOCX]

| **Supplemental Table 1. Categorization of discharge disposition in a cohort of technology-dependent children** | |
| --- | --- |
| **Mapped Value** | **Original Value (PHIS Disposition Title)** |
| Healthcare facility | Discharged/Transferred to a Short-term General Hospital for Inpatient Care |
|  | Discharged/Transferred to a Skilled Nursing Facility (SNF) with Medicare Certification in Anticipation of Skilled Care |
|  | Discharged/Transferred to a Designated Cancer Center or Children's Hospital |
|  | Discharged/Transferred to an Inpatient Rehabilitation Facility (IRF) Including Rehabilitation Distinct Part Units of a Hospital |
|  | Discharged/Transferred to a Psychiatric Hospital or Psychiatric Distinct Part Unit of a Hospital |
|  | Discharged/Transferred to a Critical Access Hospital (CAH) |
|  | Discharged/transferred to a short term general hospital for inpatient care with a planned acute care hospital inpatient readmission |
|  | Discharged/transferred to a skilled nursing facility (SNF) with Medicare certification with a planned acute care hospital inpatient readmission |
|  | Discharged/transferred to a designated cancer center or children's hospital with a planned acute care hospital inpatient readmission |
|  | Discharged/transferred to an inpatient rehabilitation facility (IRF) including rehabilitation distinct part units of a hospital with a planned acute care hospital inpatient readmission |
|  | Discharged/transferred to a Medicare certified long term care hospital (LTCH) with a planned acute care hospital inpatient readmission |
|  | Discharged/transferred to a critical access hospital (CAH) with a planned acute care hospital inpatient readmission |
|  | Discharged/transferred to another type of health care institution not defined elsewhere, with a planned acute care hospital inpatient readmission |
|  | Discharged/Transferred to an Intermediate Care Facility (ICF) |
|  | Discharged/Transferred to Long Term Care Hospitals (LTCH) |
|  | Discharged/Transferred to a Nursing Facility Certified under Medicaid but not Certified Under Medicare |
|  | Discharged/Transferred to Court/Law Enforcement |
|  | Discharged/transferred to another Type of Health Care Institution not Defined Elsewhere in this Code List (See Code 05) |
|  | Discharged/Transferred to a Federal Health Care Facility |
|  | Hospice – Medical Facility (Certified) Providing Hospice Level of Care |
| Home | Discharged to home or self care with a planned acute care hospital inpatient readmission |
|  | Discharge to Home or Self Care (Routine Discharge) |
| Home with health services | Discharged/transferred to home under care of organized home health service organization with planned acute care hospital inpatient readmission |
|  | Discharged/Transferred to Home under Care of Organized Home Health Service Organization in Anticipation of Covered Skilled Care |
|  | Hospice - Home |
