# Supplementary material for: Social Factors Predictive of Intensive Care Utilization in Technology-Dependent Children, a Retrospective Multicenter Cohort Study
Source: Front Pediatr. 2021 Sep 13;9:721353. doi: 10.3389/fped.2021.721353 (PMC8475907; doi:10.3389/fped.2021.721353)
Supplement: Supplementary file 2 [file Table_2.DOCX]

| **Supplemental Table 2. Frequency of chronic complex conditions in a cohort of technology dependent children** | |  |
| --- | --- | --- |
| CCC Categories | Entire Cohort *n* = 20,085^1^ |  |
|  |  |  |
| Any complex chronic condition | 19,876 (99%) |  |
| Cardiovascular | 6,451 (32%) |  |
| Gastrointestinal | 17,936 (89%) |  |
| Hematologic/immunologic | 1,218 (6.1%) |  |
| Malignancy | 1,170 (5.8%) |  |
| Metabolic | 2,558 (13%) |  |
| Neurologic/neuromuscular | 6,765 (34%) |  |
| Other congenital/genetic defect | 4,715 (23%) |  |
| Renal/urologic | 2,814 (14%) |  |
| Respiratory | 6,099 (30%) |  |
| Premature/neonatal | 5,108 (25%) |  |
| Technology dependence | 18,647 (93%) |  |
| Transplantation | 187 (0.9%) |  |
| CCC, chronic complex condition | |  |
