# Supplementary material for: Social Factors Predictive of Intensive Care Utilization in Technology-Dependent Children, a Retrospective Multicenter Cohort Study
Source: Front Pediatr. 2021 Sep 13;9:721353. doi: 10.3389/fped.2021.721353 (PMC8475907; doi:10.3389/fped.2021.721353)
Supplement: Supplementary file 3 [file Table_3.DOCX]

| **Supplemental Table 3. Univariate and multivariate analysis of characteristics associated with discharge disposition in a cohort of technology-dependent children who require multiple hospital admissions within a one-year observation period** | | | | | | | | | | |
| --- | --- | --- | --- | --- | --- | --- | --- | --- | --- | --- |
| Characteristic | Univariate analysis | | | | Multivariate analysis | | | | | |
|  |  |  |  |  | Health Care Facility | | | Home with Health Services | | |
|  | Home  *n* = 13,099 | Healthcare facility  *n* = 563 | Home with health services  *n* = 1,297 | p-value | OR | 95% CI | p-value | OR | 95% CI | p-value |
| Admit age on initial visit |  |  |  | <0.001 |  |  |  |  |  |  |
| <1 month | 4,302 (33%) | 190 (34%) | 512 (39%) |  | *reference* | | | | | |
| 1-12 months | 3,454 (26%) | 118 (21%) | 325 (25%) |  | 0.82 | 0.63, 1.06 | 0.13 | 0.96 | 0.82, 1.12 | 0.6 |
| 1-2 years | 1,327 (10%) | 30 (5.3%) | 108 (8.3%) |  | 0.77 | 0.50, 1.18 | 0.2 | 0.99 | 0.78, 1.25 | >0.9 |
| 2-5 years | 1,438 (11%) | 51 (9.1%) | 131 (10%) |  | 0.92 | 0.65, 1.31 | 0.6 | 1.08 | 0.87, 1.35 | 0.5 |
| 5-11 years | 1,297 (9.9%) | 67 (12%) | 105 (8.1%) |  | 1.02 | 0.73, 1.41 | >0.9 | 0.86 | 0.68, 1.09 | 0.2 |
| >11 years | 1,281 (9.8%) | 107 (19%) | 116 (8.9%) |  | 1.14 | 0.85, 1.53 | 0.4 | 0.98 | 0.78, 1.23 | 0.9 |
| Sex |  |  |  | 0.3 |  |  |  |  |  |  |
| Male | 7,145 (55%) | 323 (57%) | 723 (56%) |  |  |  |  |  |  |  |
| Female | 5,954 (45%) | 240 (43%) | 574 (44%) |  |  |  |  |  |  |  |
| Ethnicity |  |  |  | <0.001 |  |  |  |  |  |  |
| Not Hispanic or Latino | 10,383 (79%) | 439 (78%) | 1,123 (87%) |  | *reference* | | | | | |
| Hispanic or Latino | 2,716 (21%) | 124 (22%) | 174 (13%) |  | 1.12 | 0.87, 1.45 | 0.4 | 0.75 | 0.62, 0.90 | 0.003 |
| Race |  |  |  | <0.001 |  |  |  |  |  |  |
| White | 7,955 (61%) | 279 (50%) | 805 (62%) |  | *reference* | | | | | |
| Black | 2,577 (20%) | 147 (26%) | 312 (24%) |  | 1.12 | 0.88, 1.43 | 0.3 | 1.02 | 0.87, 1.19 | 0.8 |
| Asian | 461 (3.5%) | 19 (3.4%) | 32 (2.5%) |  | 0.98 | 0.57, 1.66 | >0.9 | 0.68 | 0.46, 1.00 | 0.048 |
| Other | 2,106 (16%) | 118 (21%) | 148 (11%) |  | 1.15 | 0.87, 1.50 | 0.3 | 0.89 | 0.73, 1.09 | 0.2 |
| Median household income (% FPT) |  |  |  | <0.001 |  |  |  |  |  |  |
| $>48,678 (>200%) | 4,160 (32%) | 141 (25%) | 397 (31%) |  | *reference* | | | | | |
| $36,509-$48,678 (150-200%) | 4,075 (31%) | 165 (29%) | 438 (34%) |  | 1.07 | 0.82, 1.38 | 0.6 | 1.07 | 0.92, 1.25 | 0.4 |
| $24,339-$36,509 (100-150%) | 4,250 (32%) | 219 (39%) | 392 (30%) |  | 1.17 | 0.91, 1.51 | 0.2 | 0.94 | 0.80, 1.10 | 0.4 |
| <$24,339 (<100%) | 614 (4.7%) | 38 (6.7%) | 70 (5.4%) |  | 1.12 | 0.73, 1.72 | 0.6 | 1.12 | 0.83, 1.49 | 0.5 |
| Insurance |  |  |  | <0.001 |  |  |  |  |  |  |
| Private | 4,347 (33%) | 136 (24%) | 429 (33%) |  | *reference* | | | | | |
| Public | 8,532 (65%) | 416 (74%) | 843 (65%) |  | 1.39 | 1.10, 1.75 | 0.006 | 1.01 | 0.87, 1.16 | >0.9 |
| Other | 220 (1.7%) | 11 (2.0%) | 25 (1.9%) |  | 1.07 | 0.52, 2.20 | 0.8 | 0.94 | 0.60, 1.49 | 0.8 |
| Number of complex chronic conditions |  |  |  | <0.001 |  |  |  |  |  |  |
| 1 or fewer | 428 (3.3%) | 11 (2.0%) | 29 (2.2%) |  | *reference* | | | | | |
| 2 to 4 | 9,313 (71%) | 283 (50%) | 798 (62%) |  | 0.72 | 0.38, 1.38 | 0.3 | 1.12 | 0.75, 1.67 | 0.6 |
| 5 or more | 3,358 (26%) | 269 (48%) | 470 (36%) |  | 1.01 | 0.52, 1.96 | >0.9 | 1.45 | 0.96, 2.18 | 0.080 |
| History of prematurity/low birthweight | 2,229 (17%) | 95 (17%) | 239 (18%) | 0.4 |  |  |  |  |  |  |
| Procedure received on initial visit |  |  |  | <0.001 |  |  |  |  |  |  |
| GT | 11,223 (86%) | 269 (48%) | 992 (76%) |  | *reference* | | | | | |
| Trach | 878 (6.7%) | 102 (18%) | 117 (9.0%) |  | 1.80 | 1.35, 2.39 | <0.001 | 1.21 | 0.96, 1.51 | 0.10 |
| Both | 998 (7.6%) | 192 (34%) | 188 (14%) |  | 2.20 | 1.72, 2.83 | <0.001 | 1.34 | 1.10, 1.63 | 0.004 |
| Discharge disposition on initial visit |  |  |  | <0.001 |  |  |  |  |  |  |
| Home | 10,851 (83%) | 165 (29%) | 512 (39%) |  | *reference* | | | | | |
| Home with health services | 1,605 (12%) | 35 (6.2%) | 682 (53%) |  | 1.31 | 0.90, 1.90 | 0.2 | 8.44 | 7.42, 9.60 | <0.001 |
| Healthcare facility | 643 (4.9%) | 363 (64%) | 103 (7.9%) |  | 26.1 | 20.9, 32.8 | <0.001 | 3.14 | 2.48, 3.99 | <0.001 |
| >1 readmission | 4,848 (37%) | 176 (31%) | 560 (43%) | <0.001 | 1.33 | 1.08, 1.64 | 0.008 | 1.41 | 1.25, 1.60 | <0.001 |
| OR, odds ratio; CI, confidence interval; FPT, federal poverty threshold; GT, gastrostomy tube | | | | | | | | | | |
