# Supplementary material for: Social Factors Predictive of Intensive Care Utilization in Technology-Dependent Children, a Retrospective Multicenter Cohort Study
Source: Front Pediatr. 2021 Sep 13;9:721353. doi: 10.3389/fped.2021.721353 (PMC8475907; doi:10.3389/fped.2021.721353)
Supplement: Supplementary file 4 [file Table_4.DOCX]

| **Supplemental Table 4. Univariate and multivariate analysis of characteristics associated with hospital LOS during index admission in a cohort of technology dependent children** | | | | | | | | |
| --- | --- | --- | --- | --- | --- | --- | --- | --- |
| Characteristic | Univariate analysis | | | Multivariate analysis | | | | |
|  | OR | 95% CI | p-value | OR | 95% CI | % Change | 95% CI | p-value |
| Admit age |  |  |  |  |  |  |  |  |
| <1 month | *reference* | |  | *reference* | | | |  |
| 1-12 months | 0.41 | 0.39, 0.43 | <0.001 | 0.36 | 0.35, 0.38 | -63.6 | -65.3, -61.8 | <0.001 |
| 1-2 years | 0.17 | 0.16, 0.19 | <0.001 | 0.18 | 0.17, 0.19 | -82.1 | -83.3, -80.8 | <0.001 |
| 2-5 years | 0.2 | 0.18, 0.21 | <0.001 | 0.21 | 0.19, 0.22 | -79.2 | -80.5, -77.8 | <0.001 |
| 5-11 years | 0.22 | 0.20, 0.23 | <0.001 | 0.22 | 0.21, 0.23 | -78.1 | -79.4, -76.6 | <0.001 |
| >11 years | 0.27 | 0.25, 0.29 | <0.001 | 0.26 | 0.24, 0.28 | -74 | -75.7, -72.3 | <0.001 |
| Sex |  |  |  |  |  |  |  |  |
| Male | *reference* | |  | *reference* | | | |  |
| Female | 0.95 | 0.91, 0.98 | 0.004 | 0.97 | 0.94, 1.00 | -3 | -6.4, 0.5 | 0.091 |
| Ethnicity |  |  |  |  |  |  |  |  |
| Not Hispanic or Latino | *reference* | |  | *reference* | | | |  |
| Hispanic or Latino | 0.94 | 0.90, 0.99 | 0.015 | 0.99 | 0.95, 1.04 | -0.6 | -5.3, 4.3 | 0.8 |
| Race |  |  |  |  |  |  |  |  |
| White | *reference* | |  | *reference* | | | |  |
| Black | 1.48 | 1.41, 1.55 | <0.001 | 1.27 | 1.21, 1.33 | 26.9 | 20.8, 33.2 | <0.001 |
| Asian | 0.9 | 0.81, 0.99 | 0.037 | 1.08 | 0.98, 1.19 | 7.7 | -2.2, 19.1 | 0.14 |
| Other | 1.15 | 1.09, 1.21 | <0.001 | 1.15 | 1.09, 1.21 | 14.8 | 8.9, 21.0 | <0.001 |
| Median household income (% FPT) |  |  |  |  |  |  |  |  |
| >$48,678 (>200%) | *reference* | |  | *reference* | | | |  |
| $36,509-$48,678 (150-200%) | 1.12 | 1.07, 1.18 | <0.001 | 1.07 | 1.02, 1.12 | 6.7 | 1.9, 11.6 | 0.006 |
| $24,339-$36,509 (100-150%) | 1.23 | 1.17, 1.29 | <0.001 | 1.13 | 1.08, 1.19 | 13.3 | 8.1, 18.7 | <0.001 |
| <$24,339 (<100%) | 1.51 | 1.38, 1.67 | <0.001 | 1.22 | 1.11, 1.33 | 21.7 | 11.2, 33.4 | <0.001 |
| Insurance |  |  |  |  |  |  |  |  |
| Private | *reference* | |  | *reference* | | | |  |
| Public | 1.17 | 1.12, 1.21 | <0.001 | 1.11 | 1.06, 1.15 | 10.9 | 6.5, 15.4 | <0.001 |
| Other | 0.91 | 0.79, 1.05 | 0.2 | 1.11 | 0.97, 1.28 | 11.3 | -2.8, 28.2 | 0.13 |
| Number of complex chronic conditions |  |  |  |  |  |  |  |  |
| 1 or fewer | *reference* | |  | *reference* | | | |  |
| 2 to 4 | 1.49 | 1.34, 1.65 | <0.001 | 1.24 | 1.12, 1.37 | 23.9 | 11.8, 36.8 | <0.001 |
| 5 or more | 4.15 | 3.71, 4.62 | <0.001 | 2.34 | 2.10, 2.59 | 133.7 | 109.9, 159.5 | <0.001 |
| History of prematurity/low birthweight |  |  |  |  |  |  |  |  |
| No | *reference* | |  | *reference* | | | |  |
| Yes | 2.26 | 2.14, 2.38 | <0.001 | 1.39 | 1.32, 1.46 | 39 | 32.1, 46.4 | <0.001 |
| Procedure received |  |  |  |  |  |  |  |  |
| GT | *reference* | |  | *reference* | | | |  |
| Tracheostomy | 2.09 | 1.95, 2.24 | <0.001 | 2.66 | 2.48, 2.85 | 165.5 | 147.8, 184.9 | <0.001 |
| Both | 3.68 | 3.45, 3.92 | <0.001 | 2.67 | 2.50, 2.86 | 167.1 | 150.0, 185.6 | <0.001 |
| Discharge disposition |  |  |  |  |  |  |  |  |
| Home | *reference* | |  | *reference* | | | |  |
| Home with health services | 1.45 | 1.37, 1.53 | <0.001 | 1.12 | 1.07, 1.18 | 12.2 | 6.8, 18.2 | <0.001 |
| Healthcare facility | 1.78 | 1.66, 1.91 | <0.001 | 1.49 | 1.39, 1.59 | 49 | 39.3, 59.5 | <0.001 |
| LOS, length of stay; OR, odds ratio, CI, confidence interval; FPT, federal poverty threshold; GT, gastrostomy tube | | | | | | | | |
