# Supplementary material for: Social Factors Predictive of Intensive Care Utilization in Technology-Dependent Children, a Retrospective Multicenter Cohort Study
Source: Front Pediatr. 2021 Sep 13;9:721353. doi: 10.3389/fped.2021.721353 (PMC8475907; doi:10.3389/fped.2021.721353)
Supplement: Supplementary file 5 [file Table_5.DOCX]

| **Supplemental Table 5. Univariate and multivariate analysis of characteristics associated with 30-day hospital readmission in a cohort of technology-dependent children** | | | | | | |
| --- | --- | --- | --- | --- | --- | --- |
|  | Univariate analysis | | | Multivariate analysis | | |
| Characteristic | Not readmitted | 30-day readmission | p-value | OR | 95% CI | p-value |
|  | *n* = 13,331 | *n* = 6,754 |  |  |  |  |
| Admit age |  |  | <0.001 |  |  |  |
| <1 month | 4,191 (31%) | 2,263 (34%) |  | *reference* | |  |
| 1-12 months | 3,414 (26%) | 1,762 (26%) |  | 1.03 | 0.95, 1.11 | 0.5 |
| 1-2 years | 1,193 (8.9%) | 687 (10%) |  | 1.16 | 1.04, 1.30 | 0.008 |
| 2-5 years | 1,484 (11%) | 737 (11%) |  | 1.01 | 0.91, 1.12 | 0.9 |
| 5-11 years | 1,495 (11%) | 633 (9.4%) |  | 0.87 | 0.77, 0.97 | 0.01 |
| >11 years | 1,554 (12%) | 672 (9.9%) |  | 0.9 | 0.80, 1.00 | 0.048 |
| Sex |  |  | >0.9 |  |  |  |
| Male | 7,272 (55%) | 3,681 (55%) |  |  |  |  |
| Female | 6,059 (45%) | 3,073 (45%) |  |  |  |  |
| Ethnicity |  |  | 0.004 |  |  |  |
| Not Hispanic or Latino | 10,815 (81%) | 5,362 (79%) |  | *reference* | |  |
| Hispanic or Latino | 2,516 (19%) | 1,392 (21%) |  | 1.1 | 1.02, 1.19 | 0.018 |
| Race |  |  | <0.001 |  |  |  |
| White | 8,438 (63%) | 3,980 (59%) |  | *reference* | |  |
| Black | 2,361 (18%) | 1,465 (22%) |  | 1.23 | 1.14, 1.34 | <0.001 |
| Asian | 478 (3.6%) | 224 (3.3%) |  | 1.01 | 0.86, 1.19 | 0.9 |
| Other | 2,054 (15%) | 1,085 (16%) |  | 1.01 | 0.93, 1.11 | 0.8 |
| Median household income (% FPT) |  |  | 0.8 |  |  |  |
| $>48,678 (>200%) | 4,255 (32%) | 2,141 (32%) |  | *reference* | |  |
| $36,509-$48,678 (150-200%) | 4,174 (31%) | 2,114 (31%) |  | 0.93 | 0.86, 1.00 | 0.058 |
| $24,339-$36,509 (100-150%) | 4,310 (32%) | 2,177 (32%) |  | 0.88 | 0.81, 0.95 | <0.001 |
| <$24,339 (<100%) | 592 (4.4%) | 322 (4.8%) |  | 0.89 | 0.76, 1.03 | 0.12 |
| Insurance |  |  | <0.001 |  |  |  |
| Private | 4,947 (37%) | 2,054 (30%) |  | *reference* | |  |
| Public | 8,166 (61%) | 4,563 (68%) |  | 1.31 | 1.22, 1.40 | <0.001 |
| Other | 218 (1.6%) | 137 (2.0%) |  | 1.59 | 1.27, 1.99 | <0.001 |
| Number of complex chronic conditions |  |  | <0.001 |  |  |  |
| 1 or fewer | 448 (3.4%) | 208 (3.1%) |  | *reference* | |  |
| 2 to 4 | 9,723 (73%) | 4,584 (68%) |  | 1.02 | 0.86, 1.21 | 0.8 |
| 5 or more | 3,160 (24%) | 1,962 (29%) |  | 1.34 | 1.13, 1.61 | 0.001 |
| History of prematurity/low birthweight | 2,037 (15%) | 1,195 (18%) | <0.001 |  |  |  |
| No |  |  |  | *reference* | |  |
| Yes |  |  |  | 1.09 | 1.01, 1.19 | 0.032 |
| Procedure received |  |  | <0.001 |  |  |  |
| GT | 11,177 (84%) | 5,645 (84%) |  | *reference* | |  |
| Tracheostomy | 1,055 (7.9%) | 454 (6.7%) |  | 0.81 | 0.72, 0.91 | <0.001 |
| Both | 1,099 (8.2%) | 655 (9.7%) |  | 1.01 | 0.91, 1.13 | 0.8 |
| Discharge disposition |  |  | 0.14 |  |  |  |
| Home | 10,265 (77%) | 5,179 (77%) |  |  |  |  |
| Home with health services | 1,960 (15%) | 1,051 (16%) |  |  |  |  |
| Healthcare facility | 1,106 (8.3%) | 524 (7.8%) |  |  |  |  |
| OR, odds ratio, CI, confidence interval; FPT, federal poverty threshold; GT, gastrostomy tube | | | | | | |
