# Supplementary material for: Social Factors Predictive of Intensive Care Utilization in Technology-Dependent Children, a Retrospective Multicenter Cohort Study
Source: Front Pediatr. 2021 Sep 13;9:721353. doi: 10.3389/fped.2021.721353 (PMC8475907; doi:10.3389/fped.2021.721353)
Supplement: Supplementary file 6 [file Table_6.DOCX]

| **Supplemental Table 6. Univariate analysis of patient demographics and clinical characteristics in a cohort of technology-dependent children, presented by discharge disposition** | | | | |
| --- | --- | --- | --- | --- |
| Characteristic | Home  *n* = 15,383 | Home with health services  *n* = 3,055 | Healthcare facility  *n* = 1,647 | p-value |
| Admit age |  |  |  | <0.001 |
| <1 months | 4,685 (30%) | 1,300 (43%) | 469 (28%) |  |
| 1-12 months | 4,046 (26%) | 755 (25%) | 375 (23%) |  |
| 1-2 years | 1,572 (10%) | 228 (7.5%) | 80 (4.9%) |  |
| 2-5 years | 1,806 (12%) | 263 (8.6%) | 152 (9.2%) |  |
| 5-11 years | 1,645 (11%) | 270 (8.8%) | 213 (13%) |  |
| >11 years | 1,629 (11%) | 239 (7.8%) | 358 (22%) |  |
| Sex |  |  |  |  |
| Male | 8,345 (54%) | 1,640 (54%) | 968 (59%) | 0.001 |
| Female | 7,038 (46%) | 1,415 (46%) | 679 (41%) |  |
| Ethnicity |  |  |  |  |
| Not Hispanic or Latino | 12,154 (79%) | 2,663 (87%) | 1,360 (83%) | <0.001 |
| Hispanic or Latino | 3,229 (21%) | 392 (13%) | 287 (17%) |  |
| Race |  |  |  | <0.001 |
| White | 9,621 (63%) | 1,964 (64%) | 833 (51%) |  |
| Black | 2,707 (18%) | 696 (23%) | 423 (26%) |  |
| Asian | 555 (3.6%) | 88 (2.9%) | 59 (3.6%) |  |
| Other | 2,500 (16%) | 307 (10%) | 332 (20%) |  |
| Median household income (% FPT) |  |  |  | <0.001 |
| $>48,678 (>200%) | 5,026 (33%) | 936 (31%) | 434 (26%) |  |
| $36,509-$48,678 (150-200%) | 4,764 (31%) | 1,049 (34%) | 475 (29%) |  |
| $24,339-$36,509 (100-150%) | 4,927 (32%) | 936 (31%) | 624 (38%) |  |
| <$24,339 (<100%) | 666 (4.3%) | 134 (4.4%) | 114 (6.9%) |  |
| Insurance |  |  |  | <0.001 |
| Private | 5,467 (36%) | 1,055 (35%) | 479 (29%) |  |
| Public | 9,671 (63%) | 1,938 (63%) | 1,120 (68%) |  |
| Other | 245 (1.6%) | 62 (2.0%) | 48 (2.9%) |  |
| Number of complex chronic conditions |  |  |  | <0.001 |
| 1 or fewer | 553 (3.6%) | 77 (2.5%) | 26 (1.6%) |  |
| 2 to 4 | 11,407 (74%) | 2,010 (66%) | 890 (54%) |  |
| 5 or more | 3,423 (22%) | 968 (32%) | 731 (44%) |  |
| History of prematurity/low birthweight |  |  |  | 0.048 |
| No | 12,923 (84%) | 2,525 (83%) | 1,405 (85%) |  |
| Yes | 2,460 (16%) | 530 (17%) | 242 (15%) |  |
| Procedure received |  |  |  | <0.001 |
| GT | 13,631 (89%) | 2,439 (80%) | 752 (46%) |  |
| Trach | 904 (5.9%) | 256 (8.4%) | 349 (21%) |  |
| Both | 848 (5.5%) | 360 (12%) | 546 (33%) |  |
| FPT, federal poverty threshold; GT, gastrostomy tube | | | | |
